# Generation and Characterization of Monoclonal Antibodies against a Cyclic Variant of Hepatitis C Virus E2 Epitope 412-422

**DOI:** 10.1128/JVI.02397-15

**Published:** 2016-03-11

**Authors:** Annamaria Sandomenico, Antonio Leonardi, Rita Berisio, Luca Sanguigno, Giuseppina Focà, Annalia Focà, Alessia Ruggiero, Nunzianna Doti, Livio Muscariello, Daniela Barone, Claudio Farina, Ania Owsianka, Luigi Vitagliano, Arvind H. Patel, Menotti Ruvo

**Affiliations:** a Institute of Biostructures and Bioimaging, CNR and CIRPeB, University of Naples Federico II, Naples, Italy; b Department of Molecular Medicine and Medical Biotechnology, University of Naples Federico II, Naples, Italy; c Department of Pharmacy, University of Naples Federico II, Naples, Italy; d Kedrion SpA, Lucca, Italy; e Second University of Naples, Naples, Italy; f Medical Research Council-University of Glasgow Centre for Virus Research, Glasgow, United Kingdom

## Abstract

The hepatitis C virus (HCV) E2 envelope glycoprotein is crucial for virus entry into hepatocytes. A conserved region of E2 encompassing amino acids 412 to 423 (epitope I) and containing Trp420, a residue critical for virus entry, is recognized by several broadly neutralizing antibodies. Peptides embodying this epitope I sequence adopt a β-hairpin conformation when bound to neutralizing monoclonal antibodies (MAbs) AP33 and HCV1. We therefore generated new mouse MAbs that were able to bind to a cyclic peptide containing E2 residues 412 to 422 (C-epitope I) but not to the linear counterpart. These MAbs bound to purified E2 with affinities of about 50 nM, but they were unable to neutralize virus infection. Structural analysis of the complex between C-epitope I and one of our MAbs (C2) showed that the Trp420 side chain is largely buried in the combining site and that the Asn417 side chain, which is glycosylated in E2 and solvent exposed in other complexes, is slightly buried upon C2 binding. Also, the orientation of the cyclic peptide in the antibody-combining site is rotated by 180° compared to the orientations of the other complexes. All these structural features, however, do not explain the lack of neutralization activity. This is instead ascribed to the high degree of selectivity of the new MAbs for the cyclic epitope and to their inability to interact with the epitope in more flexible and extended conformations, which recent data suggest play a role in the mechanisms of neutralization escape.

**IMPORTANCE** Hepatitis C virus (HCV) remains a major health care burden, affecting almost 3% of the global population. The conserved epitope comprising residues 412 to 423 of the viral E2 glycoprotein is a valid vaccine candidate because antibodies recognizing this region exhibit potent neutralizing activity. This epitope adopts a β-hairpin conformation when bound to neutralizing MAbs. We explored the potential of cyclic peptides mimicking this structure to elicit anti-HCV antibodies. MAbs that specifically recognize a cyclic variant of the epitope bind to soluble E2 with a lower affinity than other blocking antibodies and do not neutralize virus. The structure of the complex between one such MAb and the cyclic epitope, together with new structural data showing the linear peptide bound to neutralizing MAbs in extended conformations, suggests that the epitope displays a conformational flexibility that contributes to neutralization escape. Such features can be of major importance for the design of epitope-based anti-HCV vaccines.

## INTRODUCTION

Hepatitis C virus (HCV), a positive-strand RNA virus belonging to the Flaviviridae family, infects nearly 3% of the world's population (1). In approximately 70 to 80% of patients, HCV establishes a chronic infection in the liver that can lead to cirrhosis, liver failure, and hepatocellular carcinoma (2). HCV exhibits a high degree of genetic variability and is classified into seven major genotypes, each of which contains a large number of related subtypes (3, 4). This diversity and the high level of intrahost variability (quasispecies) contribute to virus persistence in the infected hosts. The recently developed new therapies have profoundly improved cure rates. However, the higher costs associated with these new medications are expected to limit their wider utilization (5–7). As yet, no vaccine against the virus is available.

HCV entry into target cells is believed to be mediated by a multistep process involving the interplay of the viral envelope glycoproteins E1 and E2 and several host cell factors, such as heparan sulfate, tetraspanin CD81, scavenger receptor class B type I (SR-BI), and the tight junction (TJ) proteins claudin-1 (CLDN1) and occludin (8). E1 and E2 are transmembrane proteins with extensive N-linked glycosylation (4 and 11 N-linked glycosylation sites, respectively) consisting of a large N-terminal ectodomain and a C-terminal hydrophobic anchor (9). The ectodomain of the E2 protein contains three highly variable regions. Hypervariable region 1 (HVR1; residues 384 to 411), located at the N terminus of E2, plays an important role in HCV entry, antibody binding, and disease outcome (10). It is now well established that E2 binds CD81 and SR-BI and that these interactions are a prerequisite for virus entry (10–13). However, the precise role of the E1-E2 envelope protein complex in HCV entry is still unclear.

The viral glycoprotein E2 is the major target for neutralizing antibodies. The majority of broadly neutralizing anti-E2 antibodies isolated to date target epitopes spanning the reported CD81 binding sites of E2. Importantly, mouse monoclonal antibody (MAb) AP33 (14), rat MAb 3/11 (15), and human MAbs HCV1 (16), HC33 (17), and Hu5B3.v3 (18) block the interaction of E2 with CD81 by binding to linear epitopes located within the highly conserved E2 site encompassing residues 412 to 423, referred to as antigenic site 412 (AS412) (19) or epitope I (20). Other MAbs recognize discontinuous E2 epitopes overlapping the CD81 binding site on E2 and involving residues 395 to 424, 425 to 447, and/or 523 to 540 (21, 22). Residues 412 to 423 have been proposed to be a potential target for HCV vaccine design (18, 23–26).

Although two independent crystal structures of HCV E2 have recently been reported (27, 28), they do not provide any structural data for the region from residues 412 to 423. Indeed, the construct used by Khan and coworkers (27) spanned E2 residues 456 to 656, whereas in the structure described by Kong and coworkers (28), the N-terminal portion encompassing the region from residues 412 to 423 is disordered in the crystal. Interestingly, a peptide representing this antigenic site adopts a β-hairpin conformation when it is complexed with neutralizing MAb AP33, Hu5B3.v3, or HCV1, in which Leu413, Gln415, Gly418, and Trp420 are key residues directly involved in the hydrophobic binding surface (18, 24–26). Since the hairpin-like structure of the antibody-bound peptides suggests that the region from residues 412 to 423 adopts a similar conformation in the context of the protein, we designed and prepared a cyclic variant of the epitope to help the fragment to assume a hairpin-like structure. The cyclic peptide was used to immunize mice with a view to isolating novel anti-E2 MAbs. By a subtractive screening approach, we selected a subset of MAbs recognizing only the cyclic antigen to explore the possibility of generating AP33-like antibodies with an improved binding affinity and neutralization potency. We reasoned that such an approach might generate antibodies with the potential to more efficiently capture and lock the epitope in a conformation close to that which it putatively adopts in the context of the protein in complex with neutralizing antibodies. However, these MAbs unexpectedly failed to neutralize virus infection. The results of X-ray studies of the complex of one of these MAbs with the cyclic peptide, together with antigen-antibody reactivity data, provide possible explanations for the lack of neutralization activity and offer novel insights into the design of vaccine candidates targeting the antigenic site from residues 412 to 423.

## MATERIALS AND METHODS

### Reagents.

TRIzol was purchased from Invitrogen (Carlsbad, CA, USA). The protein G and A columns and reagents for surface plasmon resonance (SPR) were purchased from GE Healthcare. Freund's complete and incomplete adjuvant and all media, including Dulbecco's modified Eagle's medium (DMEM), Opti-MEM medium, and serum, were purchased from Gibco (Life Technologies, Italia) and Sigma-Aldrich (Milan, Italy). RPMI 1640 medium containing 10% fetal bovine serum (FBS) and 1% penicillin, streptomycin, and glutamine (PSG) was used for maintaining myeloma cells. RPMI-GM medium contained RPMI 1640 medium with 1% nonessential amino acids. For the fusion of splenocytes and myeloma cells, we used RPMI 1640 medium supplemented with 15% FBS, 2% hypoxanthine-aminopterin-thymidine (HAT), 1% PSG, and 10% hybridoma-enhancing supplement (HES). The same medium lacking HES was used for hybridoma clone selection. Reagents for peptide synthesis were from Novabiochem (Laufelfingen, Switzerland), Inbios (Naples, Italy), and GL Biochem (Shanghai, People's Republic of China). Solvents for peptide synthesis and purification were from Romil (Dublin, Ireland). Other reagents were from Sigma-Aldrich (Milan, Italy). Keyhole limpet hemocyanin (KLH) was from Pierce-Thermo Fisher (Milan, Italy). The broadly neutralizing mouse MAb AP33 has been described previously (14, 29). It was generated following immunization of BALB/c mice with a mammalian cell-expressed recombinant secretory form of the HCV genotype 1a strain Gla E1E2 lacking their respective transmembrane domains (30). Soluble E2 (sE2), comprising the region from amino acids 384 to 661 of genotype 1a strain H77 (GenBank accession no. AF009606) was expressed by infecting High Five insect cells with a recombinant baculovirus, and the protein secreted into the medium was purified by Ni-nitrilotriacetic acid chromatography.

### Peptide design, synthesis, and purification.

**FIG 1:**
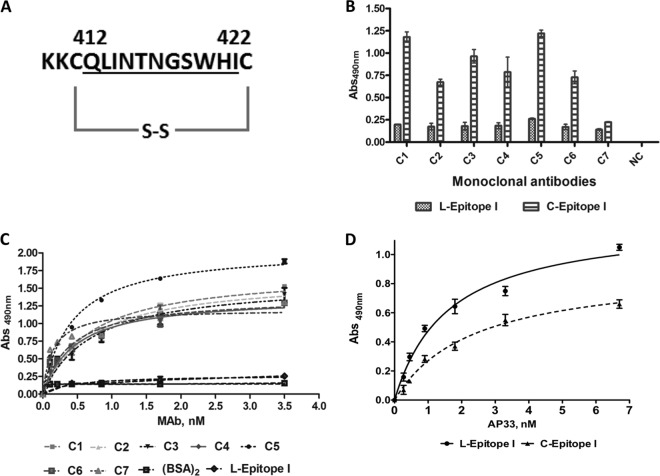
Selection of MAbs specifically binding to the cyclic variant of epitope I. (A) Primary structure of the C-epitope I peptide representing the HCV E2 region from residues 412 to 422. The peptide was C-terminally amidated, N-terminally acetylated, and cyclized using two cysteines introduced at either end of the native sequence (underlined). (B) ELISA screening of hybridoma supernatants. C-epitope I and L-epitope I were coated onto microtiter plates at 0.5 μg/ml. Hybridoma supernatants were added, and bound antibodies were detected with an HRP-conjugated anti-mouse immunoglobulin antibody. Trastuzumab was used as a negative control (NC) and was used at the same concentration as the test MAbs. (C) Dose-dependent binding of purified MAbs C1 to C7 to C-epitope I. The MAbs did not bind to L-epitope I or BSA_2_ (glutaraldehyde-self-conjugated BSA); only the negative binding data for MAb C2 are shown. (D) Binding of MAb AP33 to L- and C-epitope I peptides. Abs, absorbance.

Peptides were prepared under standard conditions of 9-fluorenylmethoxy carbonyl solid-phase synthesis (31). A cyclic peptide corresponding to the region from residues 412 to 422 of E2 (sequence, QLINTNGSWHI) was designed to facilitate the adoption of a hairpin-like conformation by the linear epitope. The structures of the complexes of the linear peptides corresponding to epitope 412-422 with MAbs AP33, Hu5B3.v3, and HCV1 (24–26) were used as a guide in the design. The peptide was generated by replacing Ile411 and Asn423 of E2 with two cysteines, which were then oxidized to form a disulfide bridge (Fig. 1A). Two lysine residues were added at the N terminus to allow conjugation to KLH and to bovine serum albumin (BSA) via glutaraldehyde (amine-to-amine cross-linking). Here this peptide is named C-epitope I. The linear variant (here named L-epitope I) used in the experiments as a control was obtained by alkylation of the cysteine thiols. Alanine-mutated variants of C-epitope I were designed and prepared under the same conditions described above to investigate the contribution of specific residues to recognition by antibodies. To overcome the structural similarity of alanine with some of the native residues, we replaced the dyad Gly417-Ser418 with glutamic acids in peptide mutant III. After synthesis and purification, peptides were cyclized as reported elsewhere (32). To suppress cysteine reactivity, the linear peptide was methylated with methyl iodide as reported previously (33). Methylation was chosen as the modification introducing the minimum structural change within the molecule.

### CD.

The purified cyclic peptide was characterized by circular dichroism (CD) using a Jasco J-710 spectropolarimeter (Jasco Corp.) equipped with a Peltier temperature control system and a 110-QS quartz cuvette with a 1.0-cm path length. The spectra of peptide solutions at 0.1 mM in 10 mM phosphate buffer, pH 7.0, were collected using the following settings: wavelength range, 190 to 280 nm; scanning speed, 20 nm/min; data pitch, 0.2 nm; bandwidth, 1 nm; and response time, 4 s. At least five independent readings of the spectra were averaged and smoothed.

### Immunogen preparation.

One milligram of C-epitope I was conjugated with 3.0 mg of carrier protein (KLH or BSA) in 2.0 ml of 20 mM phosphate buffer (pH 7.0) containing 0.2% (vol/vol) glutaraldehyde by stirring the mixture for 3 h. One milliliter of 1 M glycine in water was added to block the reaction, and then the solutions were extensively dialyzed against phosphate-buffered saline (PBS; pH 7.4) before being lyophilized. The amount of peptide-protein conjugate was determined using a Bio-Rad kit (Bio-Rad, Milan, Italy). The same procedure was used to prepare glutaraldehyde-self-conjugated BSA (BSA_2_). BSA_2_ was used as a control in enzyme-linked immunosorbent assays (ELISAs) to exclude clones producing antibodies potentially recognizing the amine-cross-linked glutaraldehyde (34).

### Immunization of mice and generation and purification of MAbs.

BALB/c mice were housed and handled according to institutional guidelines (project identification code 2013/0038120, approved by the Ethical Animal Care and Use Committee of the University of Naples Federico II; date of approval, 24 April 2013). Four 5-week-old BALB/c mice (The Jackson Laboratory) were immunized with 100 μg of KLH-conjugated peptide emulsified with complete Freund's adjuvant. Four independent injections were carried out subcutaneously with 25 μl immunogen. Before immunization, 250-μl blood samples were taken from the caudal vein of each mouse and used as the preimmune control (time zero samples). Mice were boosted with the same amount of immunogen in incomplete Freund's adjuvant at day 30 after the first immunization. Blood samples were taken from the caudal vein (250 μl) before every subsequent boosting and tested by ELISA to monitor the antibody titer. A final antigen boost was administered intravenously to mice showing the highest antibody titer 20 days before they were sacrificed and splenectomized, as described below.

Cells harvested from the spleens of sacrificed animals were fused with myeloma SP2/0 cells (ATCC) at a ratio of 5:1 in RPMI-GM containing polyethylene glycol (PEG) 1300-1600 (Hybri-Max; Sigma-Aldrich, Milan, Italy) and 7.5% dimethyl sulfoxide (Sigma-Aldrich, Milan, Italy) as described previously (35). The fused hybridoma cells were resuspended in 30 ml of selection medium consisting of RPMI-GM medium containing PEG 1300-1600, 10% FBS, 100 U/ml penicillin, 100 μg/ml streptomycin, 100 μM hypoxanthine, 16 μM thymidine, and 400 nM aminopterin (RPMI-HAT; Sigma-Aldrich, Milan, Italy). The cell suspension was dispensed into 96-well plates and incubated at 37°C in a 5% CO_2_ atmosphere with periodic replenishment with fresh selection medium. After 12 to 14 days, the cell medium was screened by ELISA for binding to C-epitope I and to its linear analogue. Hybridomas secreting antibodies with strong reactivity with C-epitope I (but not L-epitope I) were recloned twice by limiting dilution, and their reactivity was reconfirmed by ELISA. Subcloned hybridoma cells were cultured in Opti-MEM medium containing 10% FBS, gradually adapted to serum-free cell culture medium, and then transferred to bioreactors (Integra Biosciences AG, Chur, Switzerland) for large-scale antibody production. The antibodies were purified to homogeneity by protein G affinity chromatography followed by gel filtration. Antibodies were characterized by SDS-PAGE, analytical size-exclusion chromatography, and Western blotting. The immunoreactivity of purified antibodies with antigen was determined by ELISA, as described below.

### Fab fragment generation and purification.

Briefly, Fab fragments were prepared by papain digestion of purified IgGs. The reaction, monitored by SDS-PAGE, was optimized in 20 mM sodium phosphate and 10 mM EDTA (pH 7.0) buffer using papain at a 1:100 (wt/wt) ratio (Sigma-Aldrich, Milan, Italy) for 3 h at 37°C. The Fc portion was removed using a HiTrap protein G column (GE Healthcare, Milan, Italy), and then the Fab fragment was further purified by gel filtration on a Sephadex 75 column (GE Healthcare, Milan, Italy) in PBS or 25 mM Tris-HCl, 100 mM NaCl, pH 7.5. The concentration and purity of the antibodies and Fab fragments were estimated by determination of the absorbance at 280 nm using a NanoDrop 2000 spectrophotometer, SDS-PAGE, and size-exclusion high-performance liquid chromatography.

### ELISAs.

Titrations of antibody in mouse serum and screening of hybridoma supernatants were performed by ELISA as described elsewhere (36). Hybridomas were screened for their ability to secrete MAbs specific for the cyclic antigen. For this purpose, supernatants were tested by coating plates with both BSA-conjugated C-epitope I and BSA-conjugated L-epitope I. Also, BSA_2_ was used as a control to exclude clones producing antibodies recognizing the BSA-linked glutaraldehyde. Positive clones were stabilized by 3 sequential rounds of limiting dilution passages in 96-well plates. Following incubation for 2 weeks at 37°C under 5% CO_2_, the supernatant from each well was again tested for the presence of antibodies against the peptide antigen. Hybridomas secreting antibody at the highest titer were further subjected to 3 rounds of limiting dilution cloning. Finally, the antibodies secreted by selected hybridomas were purified and tested by ELISA for reactivity to the free C-epitope I and its linear variant. Briefly, the wells of ELISA plates (MaxiSorp; Nunc) were coated with the appropriate peptide at 0.5 μg/ml in PBS by incubation overnight at 4°C. The wells were blocked with BSA and then washed with PBS-Tween 20 as described above. Antigen-coated wells were incubated with 0.5 μg/ml of appropriate antibodies at 37°C for 60 min, washed as described above, and then further incubated with horseradish peroxidase (HRP)-conjugated anti-mouse IgG (1:1,000; Bio-Rad) at 37°C for 60 min. After washing, the bound antibody was detected by adding peroxidase substrate solution (prepared by dissolving *o*-phenylenediamine dihydrochloride at 0.4 mg/ml in 0.1 M citric acid and 0.2 M Na_2_HPO_4_ buffer [pH 4.8] and adding 0.2 μl/ml of 30% H_2_O_2_). Following incubation at room temperature in the dark, the reaction was stopped with a 2.5 M H_2_SO_4_ solution, and the optical density at 492 nm was determined using a microplate reader (BioTek, Winooski, VT, USA). Specificity was assessed using an unrelated monoclonal antibody (trastuzumab; Genentech) as a negative control (NC) and MAb AP33 as a positive control. The negative and positive controls were used at the same concentration as the test MAbs.

An ELISA to detect MAb binding to mammalian cell-expressed sE2, full-length (FL) E2, and the E1E2 glycoprotein was performed essentially as described previously (14, 37). Briefly, HEK293T cells were cotransfected with plasmids containing the appropriate sequence, and the expressed glycoproteins present in the clarified lysates of these cells were captured on Galanthus nivalis agglutinin (GNA)-coated Immulon II enzyme immunoassay (EIA) plates (Thermolab Systems). Anti-E2 MAbs were added, and bound glycoproteins were detected with an anti-mouse immunoglobulin G-horseradish peroxidase (Sigma, United Kingdom) and 3,3,5,5-tetramethylbenzidine (TMB; Sigma, United Kingdom) substrate. Absorbance values at 450 nm were determined. Assays for the screening of the clones were performed at least twice in quadruplicate. Data are reported as the average of the results from all experiments and replicates ± standard deviation (SD). Assays for MAb binding to peptides and recombinant proteins were performed at least twice. Data are reported as the average of the results from all experiments and replicates ± SD.

### Sequencing of antibody variable regions and isotype determination.

To clone the Ig heavy (H)-chain and light (L)-chain variable-region genes encoding a subset of the anti-E2 MAbs, total RNA from hybridoma cells was first used to generate cDNA libraries by reverse transcription reactions with a SuperScript III first-strand kit (Invitrogen) using random hexamers. The IgG Fab fragments corresponding to the antigen-binding variable regions were then amplified by PCR using a set of primers specific for mouse Ig heavy and light chains. The nucleotide sequences of the PCR products were determined, and their amino acid sequences were deduced using the program Translate from the ExPASy proteomic server. The isotypes of the MAbs were determined using a monoclonal antibody isotyping kit (IsoStrip; Pierce, Rockford, IL, USA) according to the manufacturer's instructions.

### SPR analysis.

All SPR analyses were performed on a Biacore 3000 instrument from GE Healthcare, using CM5 sensor chips and the HBS buffer certified for use with this instrument (20 mM HEPES, 0.15 M NaCl, pH 7.2, 0.005% polysorbate 20), at 25°C. Immobilization was carried out by means of the canonical amine-coupling chemistry using the surface immobilization wizard procedure operating at 5 μl/min. Channels were activated with a mixture of 1-ethyl-3-(3-dimethylaminopropyl)-carbodiimide and *N*-hydroxysuccinimide for 7 min; then, the ligand, which had been appropriately diluted in the preselected sodium acetate buffer, was coupled until a typical level of 500 to 600 response units (RU) was achieved. The remaining active ester groups were blocked with 1 M ethanolamine HCl, pH 8.5. Measurements of SPR binding to L-epitope, C-epitope I, and the alanine-mutated variants were carried out by immobilizing each antibody or Fabs on the CM5 sensor chips. Immobilization of the antibodies, including AP33, was efficiently performed at 5.0 μg/ml in 10 mM sodium acetate, pH 4.5, achieving immobilization levels of about 500 to 600 RU in every case. The same procedure was applied to prepare an sE2-functionalized sensor chip. An IgG1 isotype-matched antibody was used as a negative control at a concentration of 5.0 μM. For epitope mapping, the Fab fragment of the C2 antibody (Fab C2) at 5.0 μg/ml in 10 mM sodium acetate, pH 4.5, was directly immobilized onto the CM5 sensor chip (immobilization level, about 500 to 600 RU). An underivatized surface was prepared on every sensor chip and used as a blank control. All analyses were carried out at a flow rate of 20 μl/min, and a constant volume of 60 μl of protein or peptide solution appropriately diluted in the HBS running buffer was injected.

For every analysis, experimental sensorgrams were aligned, the blank signal was subtracted, and the sensorgrams were overlapped. All mathematical manipulations and fitting of the data were performed using BIAevaluation (version 4.1) software (GE Healthcare). All experimental data gave optimal fittings when they were processed by assuming a 1:1 Langmuir binding interaction.

### HCVcc neutralization assays.

Cell culture-infectious HCV (HCVcc) was produced by electroporation of Huh7 human hepatoma cells with viral genomic RNA that was generated by *in vitro* transcription using plasmid pUC-JFH1 or pUC-JFH1-N417T as the template as described previously (38, 39). Virus neutralization assays were performed using Huh7-J20 reporter cells, and virus infectivity levels were determined by a secreted alkaline phosphatase (SEAP) reporter assay, as described previously (40). Briefly, Huh7-J20 cells were plated out at a density of 5 × 10^3^ per well in a 96-well plate. Virus was preincubated with the test antibody at 37°C for 1 h, prior to infection of the cells at a multiplicity of infection of 0.1. At 3 h postinfection, the inoculum was replaced with fresh DMEM. At 72 h postinfection, the SEAP reporter activity (which correlates directly with the virus infectivity level) in the medium of infected cells was determined as described previously (40).

### Crystallization and diffraction data collection.

Trials of the crystallization of the complex consisting of Fab C2 and the peptide were set up at 293 K using the hanging-drop vapor diffusion method. The peptide and the Fab were previously mixed at a molar ratio of 4:1. Preliminary screenings of the crystallization conditions were carried out using commercially available sparse-matrix kits (Crystal Screen I/II and Index kits; Hampton Research) (41). These screenings yielded microcrystals that were optimized by fine-tuning of the protein and precipitant concentrations. Crystals suitable for crystallographic investigations were obtained using a Fab concentration of ∼5.0 mg/ml and 0.2 M ammonium sulfate and 25% (wt/vol) PEG 3350 in a buffer containing 0.1 M bis-Tris, pH 5.5.

Diffraction data were collected in-house at 100 K using a Rigaku Micromax 007 HF generator that produces Cu Kα radiation and that is equipped with a Saturn944 charge-coupled-device detector. Data were collected at 100 K by adding to the precipitating solution a solution of 20% (vol/vol) ethylene glycol as a cryoprotectant. The data set was scaled and merged using the HKL2000 program package (42). Although two angles of the unit cell were numerically close to 90°, the crystals of the complex were triclinic. Indeed, all attempts to process the data at a higher symmetry yielded very high *R*_merge_ values. The analysis of the Matthews coefficient (*V_m_*) value of this crystal suggests the presence of two molecules in the asymmetric unit.

### Crystallographic refinement.

The structure of the complex was solved by molecular replacement using phaser crystallographic software (43). Starting models for the light and the heavy chains were selected by looking for structures in the Protein Data Bank (PDB) with the highest sequence identities with the sequences of the chains of our Fab. Using this approach, the starting models for the heavy and light chains were extracted from the structures with PDB accession numbers 2VL5 (identity, 82%) and 3DGG (identity, 94%), respectively. Taking into account the variability of the relative orientation of the constant and variable regions, both the heavy and the light chains were fragmented by considering the individual Fab domains. Therefore, since the asymmetric unit contains two independent molecules, an ensemble of eight individual fragments constituted the starting structures in the molecular replacement search. The application of this procedure provided a straightforward solution. This model was used for automatic rebuilding, which was carried out using the ARP-wARP model-building tool (44). Crystallographic refinement was carried out against 95% of the measured data using the ccp4i program suite. The remaining 5% of the observed data, which was randomly selected, was used for *R*_free_ calculations to monitor the progress of refinement. Noncrystallographic restraints were applied in the Refmac macromolecular refinement program (45) with medium restraints for the main chain atoms and loose restraints for side chain atoms. Manual modeling was performed using the Coot tool (46). Water molecules were incorporated into the structure in several rounds of successive refinements.

### MD studies.

In order to gain insights into the intrinsic conformational properties of the cyclic peptide, a molecular dynamics (MD) simulation was conducted using the structure of the peptide detected in the complex with Fab C2 as the starting model. The molecular dynamics simulation was performed using the GROMACS software package (version 4.5.5) (47), the AMBER99sb force field, and TIP4P as the water model. The peptide was immersed in a cubic box of 4.50 by 4.50 by 4.50 nm^3^ containing 2,933 water molecules. The simulation was run with periodic boundary conditions. The temperature and pressure of the systems were stabilized at 300 K and 1 atm, respectively. Energies were minimized by fixing the protein atoms, and then the simulation was run without restraints. The time scale of the simulation was 250 ns with a time step of 0.002 ps. The particle mesh Ewald (PME) method (grid spacing, 0.12 nm) was used to calculate the electrostatic interactions. A cutoff of 10 Å was applied to treat Lennard-Jones interactions. Bond lengths were constrained using the Linear Constraint Solver (LINCS) algorithm. Trajectories were checked to assess the quality of the simulation using GROMACS routines. H-bond interactions were identified on the basis of the cutoffs for the hydrogen donor-acceptor angle (∼30°) and the donor-acceptor distance (∼3.5 Å) by using GROMACS utilities (47).

### Protein structure accession number.

The coordinates of the model and the experimental structure factors of the complex have been deposited in PDB under accession number 5EOC.

## RESULTS

### Production and selection of MAbs against C-epitope I.

Hybridoma cell supernatants were directly screened against both C-epitope I and its linear variant, L-epitope I, conjugated to BSA. Hybridomas secreting antibodies able to specifically recognize the cyclic peptide were selected for further studies. This screening strategy led to the selection of seven different hybridoma clones, and these were renamed C1 to C7 (Fig. 1B). BSA_2_ was used in the subsequent tests to exclude antibodies binding to glutaraldehyde-cross-linked BSA. These data were confirmed in binding ELISAs where the free, unconjugated peptides were coated on the plate surface. The selected MAbs bound to C-epitope I in a dose-dependent fashion (Fig. 1C) but not to L-epitope I or the BSA_2_ control (Fig. 1C). As expected, an unrelated IgG1 isotype-matched MAb, used as a negative control (NC), did not recognize either peptide (Fig. 1B). MAb AP33, used as a positive control, bound to both the cyclic and the linear peptides (Fig. 1D). A more detailed characterization of peptide binding to all antibodies was performed by SPR (see below). All selected MAbs were of the IgG1 isotype with kappa light chains.

### Evaluation of affinity of MAb C1 to C7 binding to C-epitope I.

**FIG 2:**
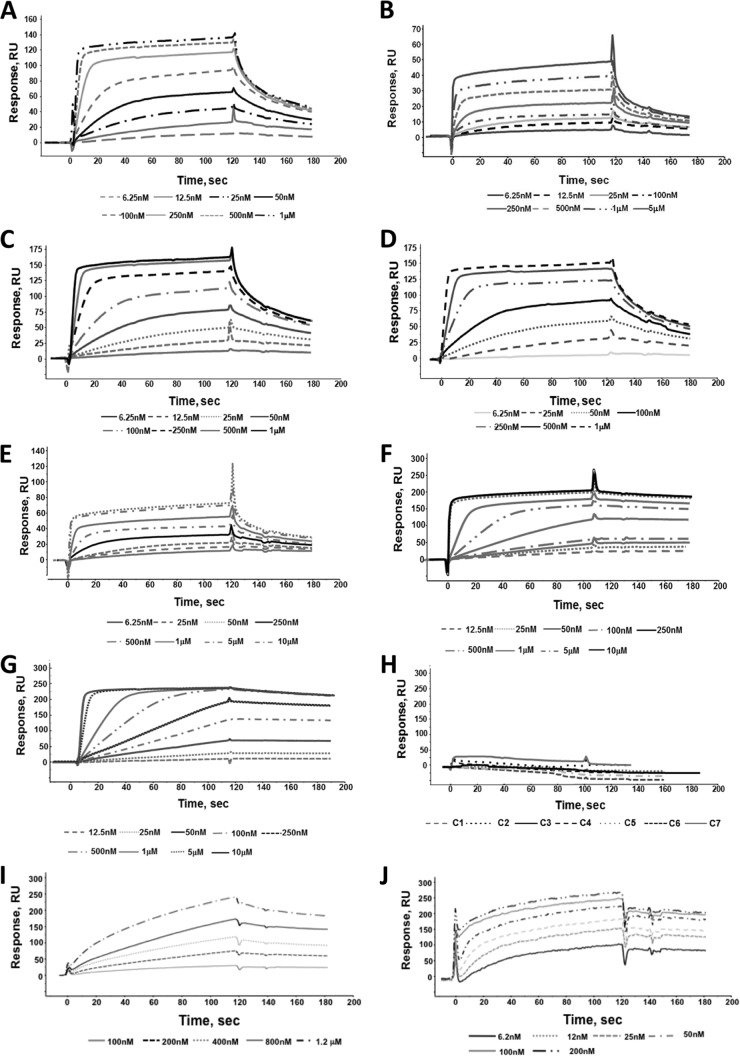
(A to G) Overlay of sensorgrams showing the binding of C-epitope I at concentrations of between 6.5 nM and 10 μM to MAbs C1 (A), C2 (B), C3 (C), C4 (D), C5 (E), C6 (F), and C7 (G) immobilized on Biacore CM5 sensor chips. (H) Overlay of sensorgrams obtained following the injection of L-epitope I at the highest concentration of 10 μM onto the 7 MAbs immobilized on Biacore CM5 sensor chips. (I and J) Overlay of sensorgrams showing the binding of C-epitope I (I) and L-epitope I (J) to MAb AP33 immobilized on Biacore CM5 sensor chips. Dose-response assays were carried out at the indicated concentrations. All experiments were carried out at 25°C and a constant flow rate of 20 μl/min using HBS as the running buffer. The values of the binding parameters are reported in Table 1.

Binding analyses were performed by SPR with the purified antibodies immobilized on distinct channels of CM5 sensor chips. SPR dose-response binding assays confirmed that only the cyclized peptide bound the MAbs with a very high affinity (Fig. 2A to G and Table 1), while no or very poor interactions were observed with the linear variant (tested at the highest concentration of 10 μM; Fig. 2H). The cyclic peptide bound all the immobilized MAbs with similar association kinetics. The dissociations were instead much slower for MAbs C6 and C7, which exhibited *K_D_* (equilibrium dissociation constant) values of about 0.9 nM and 0.7 nM, respectively (Table 1). The other MAbs showed *K_D_*s ranging from 12.7 nM to 44.2 nM. MAb AP33, raised against a recombinant soluble form of E1E2 (30), had a 150-fold higher affinity to the linear peptide than to the cyclic one (*K_D_*s = 0.5 nM and 71 nM, respectively) (Fig. 2I and J and Table 1).

**TABLE 1:** Affinity of antibodies to cyclic and linear epitope 412-422^*a*^

MAb	C-epitope I			L-epitope I		
	*K_a_* (1/M · s)	*K_d_* (1/s)	*K_D_* (M)	*K_a_* (1/M · s)	*K_d_* (1/s)	*K_D_* (M)
C1	4.84 × 10^5^	1.20 × 10^−2^	2.48 × 10^−8^			NB
C2	4.07 × 10^5^	1.23 × 10^−2^	3.03 × 10^−8^			NB
C3	3.21 × 10^5^	1.01 × 10^−2^	3.14 × 10^−8^			NB
C4	2.82 × 10^5^	1.25 × 10^−2^	4.42 × 10^−8^			NB
C5	4.34 × 10^5^	5.52 × 10^−3^	1.27 × 10^−8^			NB
C6	3.59 × 10^5^	3.22 × 10^−4^	8.97 × 10^−10^			NB
C7	3.56 × 10^5^	2.44 × 10^−4^	6.86 × 10^−10^			NB
AP33	1.21 × 10^4^	8.62 × 10^−4^	7.11 × 10^−8^	9.99 × 10^5^	5.34 × 10^−4^	5.35 × 10^−10^

a Association and dissociation rates and dissociation constants were obtained by SPR for the binding of C-epitope I and L-epitope to MAbs C1 to C7 and AP33. Data were analyzed using BIAevaluation (version 4.2) software. *K_a_*, association constant; *K_d_*, dissociation constant; NB, no binding.

### Binding of MAbs to recombinant sE2 protein.

**FIG 3:**
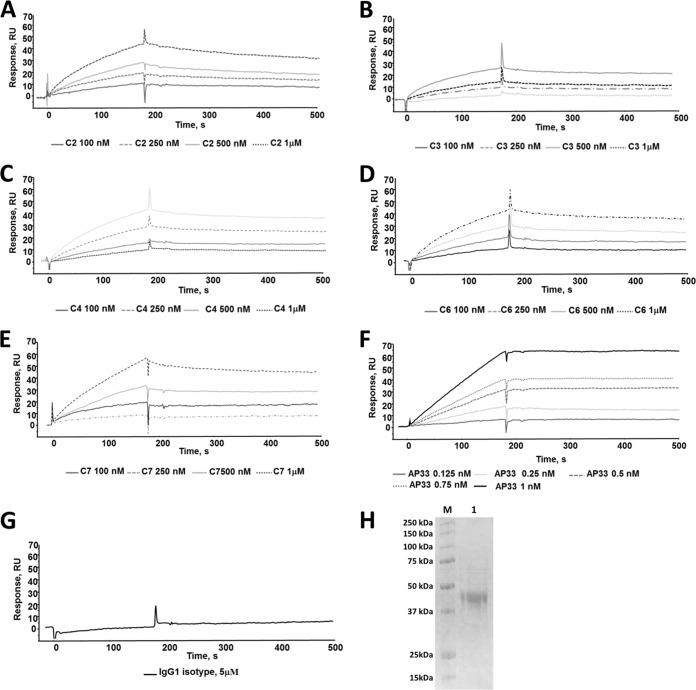
(A to G) Overlay of sensorgrams showing the dose-dependent binding of MAbs C2, C3, C4, C6, and C7 (A to E, respectively) and MAb AP33 (F) to sE2 recombinant protein immobilized on Biacore sensor chips. No interaction was detected using an IgG1 isotype at a concentration of 5 μM (G). All experiments were carried out at 25°C and a constant flow rate of 20 μl/min using HBS as the running buffer. The values of the binding parameters are reported in Table 2. (H) SDS-PAGE analysis (12% bisacrylamide) of purified sE2 used for SPR binding studies. Lane M, Precision Plus protein standards (10 to 250 kDa; Bio-Rad); lane 1, 2 μg of purified sE2 under reducing conditions. Proteins were visualized by the use of Bio-Safe Coomassie blue stain.

We next evaluated the ability of antibodies to recognize recombinant soluble E2 (sE2) (the relative purity of this protein is shown in Fig. 3H). For this purpose, dose-dependent binding assays were carried out on an sE2-functionalized CM5 sensor chip. The binding of AP33 was observed at concentrations ranging from 0.125 nM to 1 nM, while the interaction with anti-C-epitope I antibodies was observed at MAb concentrations ranging from 100 nM to 1 μM (Fig. 3A to F). In line with these observations, SPR kinetics and affinity parameters, summarized in Table 2, showed that AP33 binds sE2 with a considerably higher affinity (*K_D_* = 0.142 nM) than anti-C-epitope I MAbs, which, with the exception of C7, exhibited *K_D_* values of about 50 nM. As expected, no binding to sE2 was detected using an isotype-matched unrelated IgG1 even at concentrations as high as 5 μM (Fig. 3G).

**TABLE 2:** Affinity of antibodies to sE2^*a*^

MAb or fragment	*K_a_* (1/M · s)	*K_d_* (1/s)	*K_D_* (M)
C2	1.88 × 10^4^	8.48 × 10^−4^	49.8 × 10^−9^
C3	3.24 × 10^4^	7.35 × 10^−4^	41.6 × 10^−9^
C4	1.54 × 10^4^	5.38 × 10^−4^	44.6 × 10^−9^
C5	2.23 × 10^4^	7.31 × 10^−4^	46.8 × 10^−9^
C7	3.08 × 10^3^	2.57 × 10^−4^	83.4 × 10^−9^
AP33	6.49 × 10^5^	6.40 × 10^−5^	0.142 × 10^−9^
Fab C2	1.28 × 10^4^	4.63 × 10^−3^	301 × 10^−9^
Fab AP33	2.30 × 10^5^	1.47 × 10^−4^	0.72 × 10^−9^
IgG1 isotype control			NB

a Association and dissociation rates and dissociation constants were obtained by SPR for the binding of MAbs C2, C3, C4, C5, C7, and AP33 and unrelated mouse IgG1 to sE2. The binding of the Fab fragments of C2 and AP33 to immobilized sE2 was measured to assess the avidity effects exhibited by the full antibodies. Data were analyzed using BIAevaluation (version 4.2) software. *K_a_*, association constant; *K_d_*, dissociation constant; NB, no binding.

**FIG 4:**
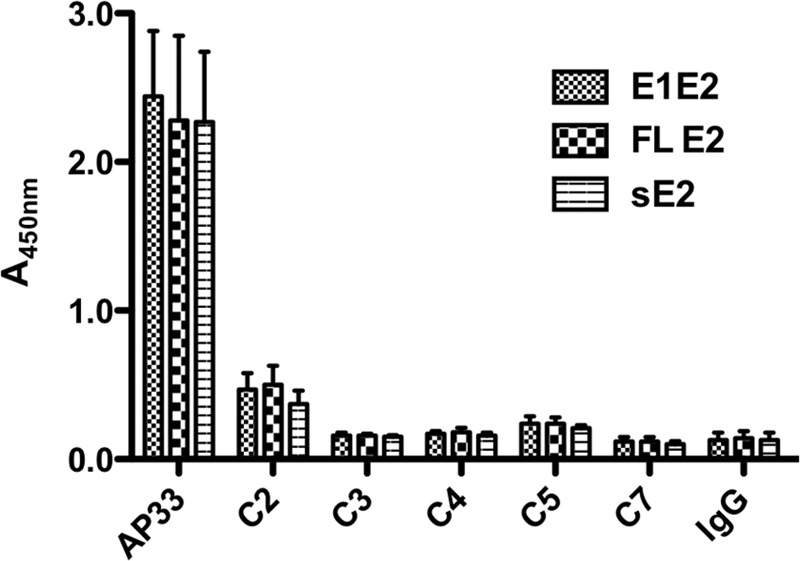
Binding of MAbs to envelope glycoproteins in ELISA. HEK293T cell lysates containing sE2, FL E2, and E1E2 were incubated on GNA-coated wells, followed by addition of MAb AP33 (0.02 μg/ml) or MABs C2 to C5 and C7 (10 μg/ml). The bound antibodies were detected using HRP-conjugated anti-mouse IgG.

Collectively, the SPR data indicate that anti-C-epitope I MAbs are able to recognize the recombinant soluble E2 protein, although their affinities are significantly lower than their affinities to the C-epitope I peptide and the affinity exhibited by AP33. It is worth noting that AP33 bound to sE2 more efficiently than it did to either the C-epitope I peptide (*K_D_*, about 71 nM; Table 1) or the linear L-epitope I peptide (*K_D_*, about 0.5 nM; Table 1). We wished to test whether MAbs could bind not only to sE2 but also to the full-length (FL) E2 protein and the E1E2 heterodimer. Transient transfection of HEK293T cells was used to produce sE2, FL E2, and E1E2. Proteins from cell extracts were captured onto GNA-coated ELISA plates. On addition of anti-C-epitope I MAbs C2, C3, C4, C5, and C7 at 10 μg/ml, a weak binding signal was observed with C2 only, while no binding was observed with the other MAbs (Fig. 4). In contrast, MAb AP33 at 0.02 μg/ml gave a strong signal with all forms of the glycoprotein (Fig. 4).

**FIG 5:**
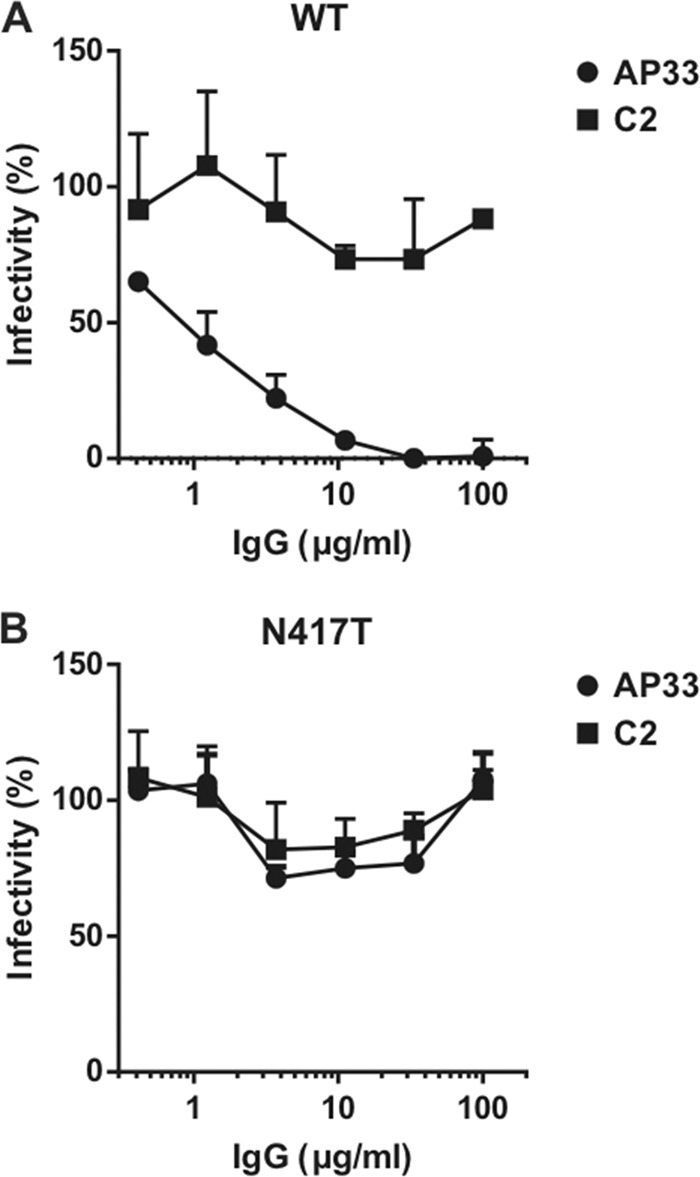
Neutralization of wild-type and N417T mutant HCVcc by MAbs AP33 and C2. Wild-type (WT) HCVcc strain JFH1 (A) or HCVcc carrying the N417T mutation (B) was incubated with a range of concentrations of MAb AP33 or C2 prior to infection of Huh7-J20 cells. At 72 h postinfection, the activity of the reporter SEAP secreted into the cell medium was measured, and the infectivity levels were plotted as the percent infectivity relative to that of a no-antibody control.

We next tested whether the MAbs were capable of neutralization of retroviral pseudoparticles bearing HCV glycoproteins (HCVpp) and HCVcc. We found that, unlike MAb AP33, all anti-C-epitope I MAbs (used at 50 and 100 μg/ml, that is, 335 and 670 nM, respectively) failed to neutralize HCVpp bearing E1E2 derived from HCV genotype 1a strain H77 (data not shown). Similarly, these MAbs were also unable to neutralize infection of Huh-7 cells with the HCVcc genotype 2a strain JFH-1 (data not shown and Fig. 5). Collectively, these data are in agreement with the lack of sE2 binding exhibited by anti-cyclic peptide MAbs, although in consideration of the concentration used in the neutralization tests and the *K_D_*s measured by SPR, they do not fully explain the complete absence of activity. Biacore binding measurements between sE2 and the Fab fragments of both C2 and AP33 showed that both MAbs displayed an average reduction of affinity of 5-fold when only one antibody arm was used (Table 2), a result suggesting that both antibodies exhibited an avidity effect.

### Crystal structure of Fab C2 in complex with C-epitope I.

**FIG 6:**
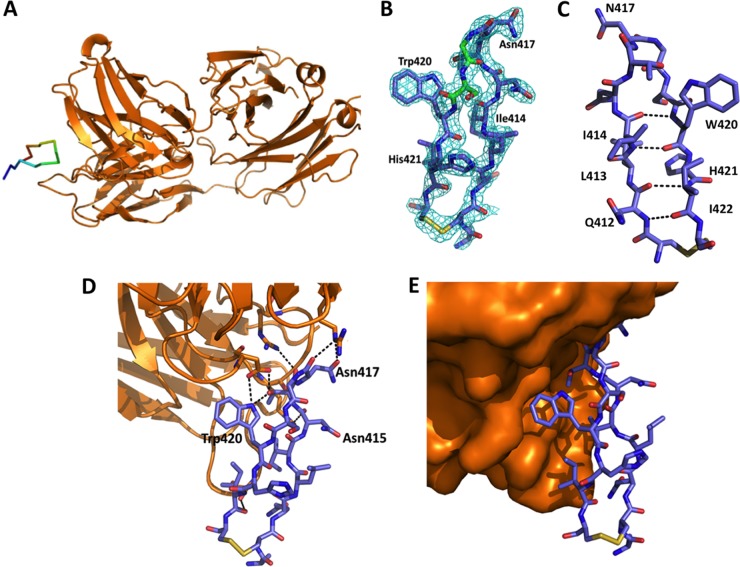
Structure of Fab C2 in complex with the C-epitope I peptide. (A) Overview of the C-epitope I peptide bound in the Fab C2 combining site. The peptide carbon atoms are ramp colored from the N terminus (blue) to the C terminus (red) through green. (B) 2*F*_o_ − *F*_c_ electron density map of the peptide region contoured at 1.0 σ. (C) Backbone intrapeptide H bonds. (D and E) H-bond (D) and hydrophobic (E) interactions at the peptide-Fab C2 interface.

To gain insights into the structural basis of the limited affinity of these MAbs for sE2 and the lack of neutralization activity, we performed crystallographic analyses of Fab complexes with the C-epitope I peptide. Although we were able to obtain crystals or microcrystals of all the Fabs bound to the C-epitope I peptide, only the Fab C2-peptide complex was suitable for crystallography. The structure of this complex was determined to a 1.98-Å resolution. The triclinic crystals used for the crystallographic investigations contained two independent copies of the complex. The structures of these two crystal mates were very similar. Indeed, the root mean square deviation (RMSD) computed on the C-α atoms was 0.58 Å. Therefore, the structural feature of molecule A (Fig. 6A) corresponding to the heavy (H) and light (L) chains was analyzed further. The Fab is composed of the canonical four immunoglobulin subunits. The elbow angles for the two Fab molecules in the asymmetric units are 129° and 131°. These values fall in the range observed for kappa Fab structures (48). The complementarity-determining region (CDR) loops of Fab C2 are also similar to those observed in canonical structures (49). Since the early stages of the refinement of the complex, the inspection of the electron density maps clearly indicated the presence of the peptide in a cleft formed by the variable regions of the light and heavy chains. As shown in Fig. 6B, the electron density is well defined for all residues of the peptide, including the two cysteine residues that form the disulfide bridge that closes the loop. The peptide adopts a β-hairpin structure that is stabilized by five hydrogen bonds, four of which involve the main chain atoms and one of which involves the side chains. Two backbone H bonds are formed by the nitrogen and the carbonyl of Gln412 with the carbonyl and the nitrogen of Ile422, respectively. A similar pair of bonds is formed by Ile414 and Trp420. The network of intrapeptide H bonds is completed by the one formed by the side chains of Trp420 (atom N-ε1) and Thr416 (atom O-γ1) (Fig. 6C).

Peptide binding by Fab C2 relies on hydrophobic interactions and the H bonds established at the antibody-combining site (Fig. 6D and E). Both the heavy and the light chains of C2 contribute to the binding. The surface areas buried upon peptide binding are 353 Å^2^ and 195 Å^2^ for the light and the heavy chains, respectively. The interactions of the light chain with the peptide involve CDRs light-chain 1 (L1) and L3. In line with other complexes of Fabs with peptides, no interactions are established by CDR L2. On the other hand, all three H-chain CDRs (CDRs H1 to H3) establish interactions with the peptide. The main hydrophobic interactions are established by Tyr36 (CDR L1), Ile95 (CDR L3), and Trp33 (CDR H1) with the nonpolar residues of the peptide (Trp420 and Ile422). Most of the H-bonding interactions involve charged side chains of the Fab. Indeed, the Arg59 (CDR H2) and Asp103 (CDR H3) side chains of the heavy chain bind Asn417 and Trp420 of the peptide, respectively. Moreover, the side chains of Arg96 and Arg100 of CDR L3 bind the carbonyl groups of the main chains of Asn415 and Thr416, respectively. The ensemble of these intermolecular H bonds is completed by the one formed by the side chain of Ser32 (CDR L1) and the carbonyl group of Ile422. Crystallographic and stereochemical statistics for the final models are summarized in Table 3.

**TABLE 3:** Data collection and refinement statistics

Parameter^*a*^	Value(s) for the peptide^*b*^
Data collection statistics	
Space group	P1
Unit cell dimensions	
*a*, *b*, *c* (Å)	54.97, 56.19, 77.15
α, β, γ (°)	90.34, 90.18, 94.98
Resolution range (Å)	25.0–1.98
No. of molecules in the asymmetric unit	2
No. of unique observations	59,746
Multiplicity	2.9 (1.9)
Completeness (%)	93.0 (81.7)
*R*_merge_	0.077 (0.476)
*I*/σ(*I*)	15.9 (1.7)
Refinement statistics	
Resolution range (Å)	15.0–19.8
*R*_factor_/*R*_free_^*c*^	0.224/0.267
No. of protein/peptide atoms	13,092
No. of water molecules	510
RMSD from ideal structure	
Bond length (Å)	0.0020
Bond angle (°)	1.30

a *R*_merge_ = Σ*_hkl_*Σ*_i_*|*I_i_*(*hkl*) − <I(*hkl*)>|/Σ*_hkl_*Σ*_i_I_i_*(*hkl*), where *I_i_*(*hkl*) is the intensity of an observation and <*I*(*hkl*)> is the mean value for its unique reflection; summations are over all reflections. *R*_factor_ = Σ*_h_*|*F*_o_(*h*) − *F*_c_(*h*)|/Σ*_h_F*_o_(*h*), where *F*_o_ and *F*_c_ are the observed and calculated structure-factor amplitudes, respectively.

b Values in parentheses refer to the 2.05- to 1.98-Å-resolution shell.

c *R*_free_ was calculated with 5% of the data excluded from the refinement.

### Molecular dynamic and circular dichroism studies.

**FIG 7:**
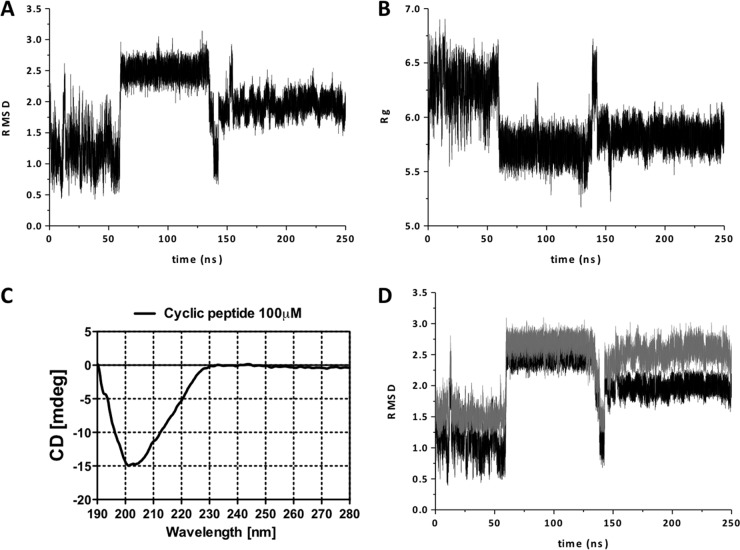
Intrinsic conformational properties of the cyclic peptide. (A) RMSD values (in angstroms) computed on the C-α atoms of the MD trajectory frames against the starting structure. (B) Time evolution of the radius of gyration (R_g_; in angstroms) computed on the C-α atoms of the peptide. (C) Far-UV CD spectrum of the peptide in phosphate buffer at neutral pH. (D) RMSD values (in angstroms) computed on the C-α atoms of the trajectory frames against the conformation of the linear peptide in the complex with AP33 (gray line; PDB accession number 4GAG). RMSD values (in angstroms) of the trajectory structures versus the starting conformation of the cyclic peptide (black) are shown for comparative purposes.

The intrinsic conformational properties of the cyclic peptide were analyzed by MD studies. These studies were conducted using the conformation of the peptide observed in the complex with Fab C2 as the starting model. The analysis of the trajectory structure clearly indicates that the peptide undergoes a significant conformational transition during the MD simulation. Indeed, as shown in Fig. 7A, the RMSDs indicate that the trajectory structures present significant variations from the structure of the starting crystallographic model. A similar behavior is highlighted by the analysis of the gyration radius of the simulation structures (Fig. 7B). The analysis of the gyration radius also indicates that the peptide frequently assumes structures that are more compact than the elongated β-hairpin motif observed for the peptide in complex with the antibody. Interestingly, these compact structures occasionally evolve into β-hairpin states resembling the crystallographic one (at approximately 140 ns, as shown in Fig. 7A). These findings indicate (i) that the cyclic peptide is intrinsically endowed with a significant level of flexibility and (ii) that the conformation detected in the complex with Fab C2 is among those conformations intrinsically accessible to the peptide.

The intrinsic flexibility of the C2 peptide was confirmed by a circular dichroism analysis performed in aqueous buffer (see Materials and Methods for details). Indeed, as shown in Fig. 7C, the CD spectrum of the peptide is suggestive of a very limited content of regular structure.

To gain deeper insights into the structural features of the conformational ensemble of the cyclic peptide, we compared the trajectory structures with the conformation adopted by the linear variant when complexed with other Fabs. Not surprisingly, when the trajectory structures were compared to the structure of the linear peptide adopting the β-hairpin motif, as in the complex with AP33, the trends were rather similar to those observed for the cyclic peptide (Fig. 7A and D). We also searched the simulation ensemble for structures showing the highest similarity with the conformation adopted by the linear variant in complex with AP33. The trajectory structures with the closest similarity presented RMSD values against this variant of 1.1 Å. Interestingly, the RMSD displayed by the conformation adopted by the cyclic peptide in the Fab C2 complex against the same peptide was larger (1.76 Å). However, although the trajectory structure was closer to that of the peptide bound to AP33, none of them perfectly reproduced it. As expected, the deviations of the trajectory structures from the elongated conformations of the peptides in complex with the Fabs of HC33.1 and 3/11 were much larger (data not shown). This is an obvious consequence of the restraints imposed by the disulfide bridge that impede the cyclic peptide from the adoption of highly extended conformations.

### C-epitope I mapping using Fab C2.

To further investigate which peptide residues were mostly involved in the binding with the antibodies, we designed and prepared a panel of alanine-mutated cyclic peptides (Table 4). This study was carried out by SPR dose-response binding assays on a sensor chip functionalized with the purified Fab fragment of C2. All peptides were tested at concentrations ranging from 125 nM to 5 μM. As shown in Table 4, we found that Fab C2 bound the wild-type C-epitope I peptide with a *K_D_* similar to that exhibited by the whole antibody (30.3 nM and 32.7 nM, respectively). When we tested the peptides bearing mutations across residues 411 to 418, we observed that the *K_D_* for peptide mutant II, where the Asn-Thr-Asn amino acids were replaced with alanines, was substantially similar to that for the wild type, that is, 14.8 nM for mutant II and 32.7 nM for the unmodified peptide. With mutants I and III, the affinity was greatly reduced (Table 4), whereas with mutant IV, bearing mutations on residues 420 to 422 (Trp-His-Ile), the binding was abolished. The data overall suggested that the triplet Trp420, His421, and Ile422 is crucial for antibody recognition. These results are in keeping with the crystallographic findings and provide quantitative information on the role played by specific residues of C-epitope I in Fab recognition.

**TABLE 4:** Binding affinity of Fab C2 to the epitope I variants used in this study^*a*^

Peptide	Sequence^*b*^	*K_D_* (M)	*K_a_* (1/s)	*K_d_* (1/M · s)
C-epitope I	KKCQLINTNGSWHIC	3.27 × 10^−8^	4.77 × 10^5^	1.56 × 10^−2^
L-epitope I	KKC(methyl)QLINTNGSWHIC(methyl)	NB	0	0
C-epitope I mutant I	KKCAAANTNGSWHIC	4.53 × 10^−7^	1.22 × 10^6^	5.53 × 10^−1^
C-epitope I mutant II	KKCQLIAAAGSWHIC	1.48 × 10^−8^	3.2 × 10^3^	4.73 × 10^−5^
C-epitope I mutant III	KKCQLINTNEEWHIC	4.67 × 10^−6^	1.62 × 10^4^	7.54 × 10^−2^
C-epitope I mutant IV	KKCQLINTNGSAAAC	NB	0	0

a Association and dissociation rates and *K_D_* values were determined by SPR for the binding of peptides to Fab C2. Data were derived using BIAevaluation (version 4.2) software. *K_a_*, association constant; *K_d_*, dissociation constant; NB, no binding.

b In C-epitope I and related mutants I to IV, a disulfide bridge connects the two cysteines. In L-epitope I, cysteines are methylated. Methylation is the minimum molecular modification required to block reactive thiols. C-epitope I mutants I, II, and IV were designed and prepared so that native residues were replace with alanines. In mutant III, glycine and serine were mutated to glutamic acid because alanines are too similar to glycine and serine.

### Neutralization of HCVcc mutant N417T by C2.

Our structural data reveal that the N417 side chain is partially buried upon Fab C2 binding (Fig. 6D). Since residue N417 is glycosylated in the native protein, we hypothesized that N417 glycosylation could be an important factor in E2 recognition by MAb C2. To further address this issue, we used a virus neutralization assay to evaluate the ability of MAb C2 to recognize an E2 variant carrying the mutation N417T, which abolishes this glycosylation site. In this mutant, the glycosylation site is shifted to N415, whose side chain is fully exposed in the structure of the complex between Fab C2 and the cyclic peptide (Fig. 6D). This glycan shift has been proposed to render E2 resistant to recognition by anti-epitope I MAbs, such as AP33 (18). As shown in Fig. 5A and B, MAb C2 failed to neutralize N417T HCVcc as well as the wild-type virus, while, as expected, MAb AP33 efficiently neutralized the wild-type virus but not the N417T mutant (18). This observation indicates that the inability of MAb C2 to recognize HCVcc is not dependent on the glycosylation status of N417.

## DISCUSSION

The elevated costs associated with current antiviral therapies against hepatitis C and the high disease prevalence necessitate the urgent development of alternative therapeutic approaches. As for many other viral diseases, a vaccine would be the most obvious and a less expensive option. However, the high genetic variability of the virus is a major barrier that has so far prevented the generation of effective anti-HCV vaccines. Several studies have indicated that the HCV surface glycoprotein E2 is a major target for neutralizing antibodies. However, they are generally isolate specific and do not recognize E2 proteins from other HCV genotypes, thus preventing their use as broad-spectrum neutralizing reagents. The conserved region from residues 412 to 422 of E2, encompassing Trp420, a key residue for HCV recognition by the human receptor CD81 (9, 11), is recognized by several neutralizing antibodies (13–17). Despite the crucial role that the residues of this epitope play in HCV entry, no information on the conformation(s) that this region adopts in the context of the E2 protein is available. Structural studies on complexes of the E2 peptide from residues 412 to 423 with three neutralizing MAbs have shown a tendency of this fragment to adopt a β-hairpin conformation (18, 24–26). In this framework, to gain insights into epitope 412-422 conformational preferences, we generated and characterized a set of MAbs that selectively recognize a conformationally restrained cyclic variant of this epitope but not its linear counterpart. Binding assays demonstrated that such MAbs were able to bind the soluble E2 protein with *K_D_*s of about 50 nM. This finding holds an interesting implication. Considering that the MAbs are unable to recognize the linear peptide representing the sequence of epitope I, their ability to bind E2 suggests that the protein context has an impact on the epitope structure, likely shifting its conformation toward bent states. However, the affinity for E2 exhibited by these MAbs is significantly lower (approximately 500-fold) than that exhibited by the neutralizing MAb AP33, and this feature largely contributes to the lack of activity of the new antibodies.

A crystallographic analysis of the complex between the Fab fragment of the C2 MAb and the cyclic peptide was undertaken to unravel the basis of the reduced affinity of the MAbs for the E2 protein and of the consequent inability to neutralize either HCVpp or HCVcc. A comparison of the crystal structure of the C-epitope I–Fab C2 complex with the structure of the linear peptide bound to the Fab fragment of hu5B3.v3, AP33, or HCV1 (18, 24–26) shows both analogies and differences. First, in all of these complexes, the peptide adopts a β-hairpin conformation stabilized by H bonds established with main chain atoms. Moreover, these peptide-Fab complexes feature a similar buried surface area (∼250 Å^2^), with an important contribution to intermolecular interactions being provided by the side chain of Trp420 (Fig. 6D and E) (18, 24–26). These observations indicate that the C2 MAb, despite its inability to neutralize virus infection, shares with hu5B3.v3, AP33, and HCV1 two important features related to the overall peptide conformation and to the tight binding to the Trp420 side chain.

**FIG 8:**
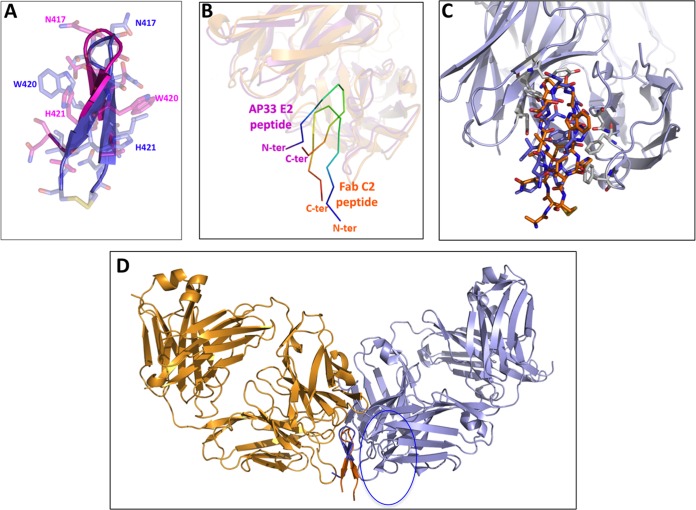
Comparison of the conformations adopted by epitope 412-422 peptides in complex with Fabs. (A) A structural alignment of peptides bound to AP33 (blue) and to Fab C2 (magenta) shows similar β-hairpin conformations. (B) The crystal structures of Fab C2 (orange; this study) and the Fab of AP33 (purple; PDB accession number 4GAG) are superimposed and faded out. The peptide carbon atoms are ramp colored from the N terminus (blue) to the C terminus (red) through green, to show that the two peptides are bound in opposite orientations relative to the Fab fragment. (C) Superimposition of the cyclic peptide (with the carbon atoms in orange) to the linear peptide in its complex with AP33. (D) The peptide structures in the complexes with Fab C2 (orange) and the Fab of AP33 (purple) are aligned to show how the two antibodies approach the opposite surfaces of the peptide hairpin-like structure.

**FIG 9:**
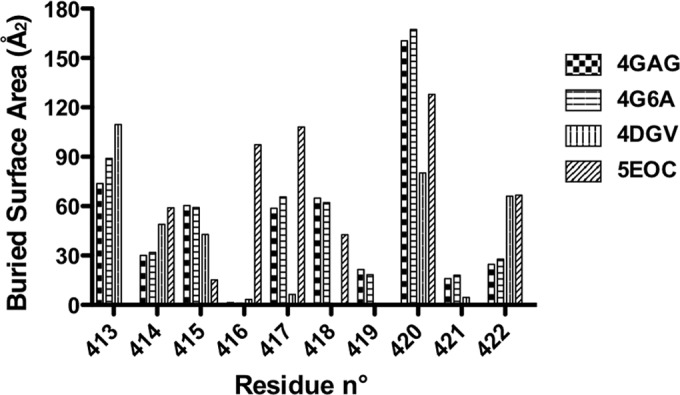
Histogram showing the buried area for residues 412 to 422 in different peptide Fab complexes. 4GAG and 4G6A correspond to the PDB accession numbers of two independent characterizations of the complex between AP33 and the linear epitope (24, 25). 4DGV is the PDB accession number for the complex between HCV1 and the linear peptide (26); 5EOC is the PDB accession number of the structure described in this work (Fab C2).

A deeper comparison of these complexes underlines, however, some distinct features in the recognition of the cyclic peptide by C2. In particular, the binding of C-epitope I by Fab C2 appears to be rotated by 180° compared to the orientations of the other complexes (Fig. 8A to D). This leads to differences in the interacting surfaces between the linear and cyclic variant in the complex with their MAbs (Fig. 9). In particular, residues such as Leu413 and Asn415, which interact with AP33 and HCV1, are fully exposed in the C2 complex. On the other hand, the side chain of Asn417, which is glycosylated in E2 and exposed to the solvent in the other complexes, is slightly buried upon C2 binding (Fig. 6D). It is worth mentioning that the failure of C2 to neutralize variants such as N417T, which cannot be glycosylated at position 417 (18), suggests that the partial burying of Asn417 does not play a major role in determining the distinctive behavior of C2.

Finally, there is a slight shift in the residues involved in the hydrogen bonding patterns of the hairpin in the peptide-C2 complex compared to the residues involved in the other complexes. The previously reported structures show that the hairpin is stabilized by H bonds between residues 414 and 421 and between residues 412 and 423, whereas the conformation of the cyclic peptide bound to C2 is stabilized by H bonds between residues 412 and 422 and between residues 414 and 420.

Predictive analyses, carried out using the PEP-FOLD server (http://bioserv.rpbs.univ-paris-diderot.fr/services/PEP-FOLD/), suggest that the structure observed for the cyclic peptide in the complex is similar to the conformation intrinsically accessible to the linear sequence of epitope 412-422. Indeed, the RMSD values of the cyclic peptide structure against the top two solutions provided by the PEP-FOLD server are in the range of 1.9 to 2.0 Å (50).

Together, these observations may in principle explain the reduced affinity of C2 MAb for E2 and suggest that the lack of neutralization may originate from a combination of a low antibody affinity and an inappropriate approach to the epitope. Indeed, despite the analogies with other complexes, the specificities of the C2 complex to the cyclic variant in terms of peptide H-bond patterns and side chain conformations, as well as in terms of the relative orientation of the epitope and MAb, may limit the ability of C2 to recognize E2. This implies that the conformations of the linear peptides observed in the complexes with HCV1 and AP33 represent a reliable model of the structure of the epitope in the context of real E2.

In this framework, the absence of a significant neutralizing effect of the MAbs generated against the cyclic variant is likely due to the inability of this peptide to fully reproduce the epitope conformation of the linear variant observed in the complexes with AP33 and HCV1, which was supported by our MD analysis. However, it should be noted that while the manuscript was in preparation, two novel complexes of neutralizing Fabs of MAbs 3/11 and HC33.1 with the linear epitope were reported (51, 52). Surprisingly, in both of these new complexes, the bound peptides assumed rather extended conformations that were completely unrelated to those observed in the complexes with AP33 and HCV1. These new data strongly suggest that epitope 412-422 is, in the protein context, endowed with a remarkable structural versatility, a property believed to be an additional mechanism of neutralization escape (51, 52). The observation that the epitope conformation recognized by the Fabs of 3/11 and HC33.1 may differ from that recognized by the Fabs of AP33 and HCV1 suggests that the specificities of the conformation of the cyclic peptide recognized by C2 may not be the only factor responsible for the inactivity of this MAb.

It is likely that our MAbs, which recognize with high selectivity a conformationally restrained variant of epitope 412-422, are unable to accommodate all of its accessible structural states. In other words, these MAbs are able to bind only a subpopulation of the diverse conformational ensemble adopted by epitope 412-422, a property that reflects the reduced affinity of our MAbs for the E2 protein and their inability to display any significant neutralizing activity. On the other hand, neutralizing MAbs such as AP33, which recognize both cyclic and linear variants of epitope 412-422, have a high affinity for E2 since they are able to capture different conformational states of this E2 region. These considerations should be taken into account in the selection of HCV-neutralizing MAbs and for the design of new potential vaccines.

In conclusion, our data corroborate the emerging notion that epitope 412-422 is characterized within the protein context by a high degree of conformational versatility which likely contributes to a mechanism of conformation-driven neutralization escape. Since rigid and flexible regions in a protein are typically characterized by conserved and variable sequences, respectively, further studies are needed to clarify why and how the E2 epitope 412-422 region combines a structural flexibility with a highly conserved local sequence.
